# First person – Tamio Furuse and Hiroshi Mizuma

**DOI:** 10.1242/dmm.042127

**Published:** 2019-09-01

## Abstract

First Person is a series of interviews with the first authors of a selection of papers published in Disease Models & Mechanisms (DMM), helping early-career researchers promote themselves alongside their papers. Tamio Furuse and Hiroshi Mizuma are co-first authors on ‘ A new mouse model of GLUT1 deficiency syndrome exhibits abnormal sleep-wake patterns and alterations of glucose kinetics in the brain’, published in DMM. Tamio is a research and development scientist in the lab of Masaru Tamura at RIKEN BioResource Research Center, Japan, investigating the development of a new phenotyping platform of mutant mice. Hiroshi is a research scientist in the lab of Yasuyoshi Watanabe at RIKEN Center for Biosystems Dynamics Research, Japan, investigating functional brain PET imaging in mice modelling human disease.


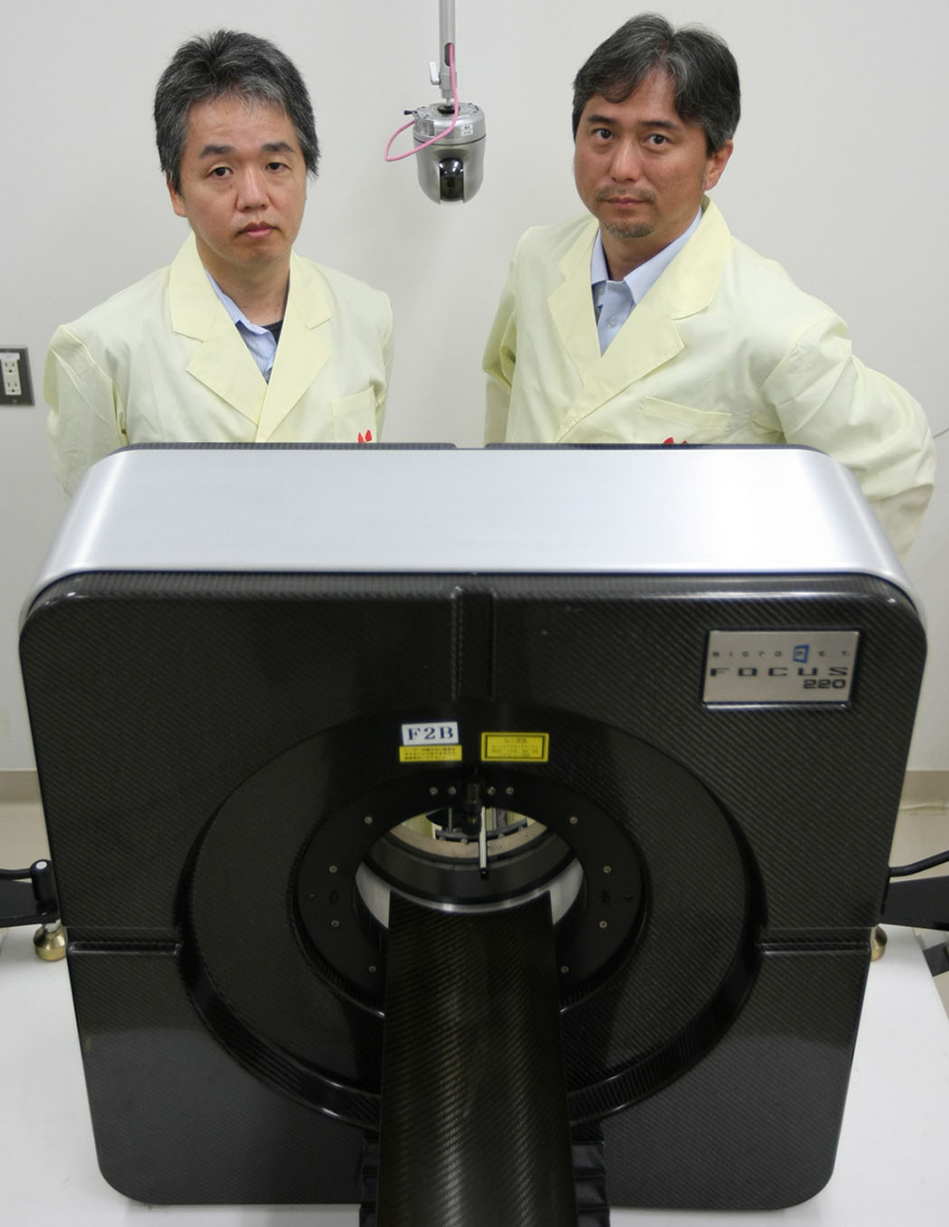


**Tamio Furuse and Hiroshi Mizuma**

**How would you explain the main findings of your paper to non-scientific family and friends?**

TF: GLUT1 deficiency syndrome (GLUT1DS), an intractable form of epilepsy, is caused by dysfunction of glucose transporter 1, which delivers glucose to the brain. Traditional anticonvulsants are not effective for GLUT1DS. In order to develop new drugs and therapeutics, animal models of GLUT1DS are necessary. As such, our findings may help patients with intractable epilepsy, GLUT1DS, by providing a good model for the disease.

HM: In this study, we performed 18F-FDG PET, with the aim of understanding brain glucose kinetics in a GLUT1DS mouse model. PET imaging has the main advantage of enabling quantitative, non-invasive, *in vivo* measurement of molecular functionality. To our knowledge, this is the first study to measure, quantitatively, brain glucose kinetics in the GLUT1DS mouse model under non-anesthetized conditions. In the future, we hope that our findings will contribute to new aspects of clinical investigation in patients with GLUT1DS.

“To our knowledge, this is the first study to measure, quantitatively, brain glucose kinetics in the GLUT1DS mouse model under non-anesthetized conditions” – *Hiroshi Mizuma*

**What are the potential implications of these results for your field of research?**

TF: ENU mutagenesis, a traditional methodology, is still able to provide a useful insight into disease models and could be harmonized with phenotyping methods based on advanced technology such as PET imaging.

HM: We have developed a methodology for brain PET imaging in mice under non-anesthetized conditions. By using this, we can gain insight into intrinsic functioning of the brain during near-physiological conditions. We have measured brain glucose metabolic activity not only in this GLUT1DS mouse model but also in other mouse models of human disease, such as dementia and autistic spectrum disorder. Our imaging method also enabled the detection of regional abnormal glucose metabolism in these mouse models.

**What are the main advantages and drawbacks of the model system you have used as it relates to the disease you are investigating?**

TF: The main advantage is that brain structures are conserved between human and mice. A drawback is that advanced social behavior in humans, such as the use of language, are not reproduced in mice.

**What has surprised you the most while conducting your research?**

TF: The founder mouse of the *Glut1* mutant line exhibited visible seizures. At the beginning of genetic analysis of the mutant, I predicted that the mutated gene was expressed in neurons or glia which directly control brain function. However, the *Glut1* gene is expressed in the blood-brain barrier, not in neurons.

HM: Although we were concerned about whether our PET imaging method could be used to obtain data of glucose kinetics in a mouse as in human PET studies, our results indicate the hypofunction of glucose transport in the brain in GLUT1DS model mice. We were surprised that this mouse model showed an increase in the uptake of FDG in the brain, resulting in a compensatory upregulation of intracellular high glucose phosphorylation against low glucose transporter function.

**Figure:**
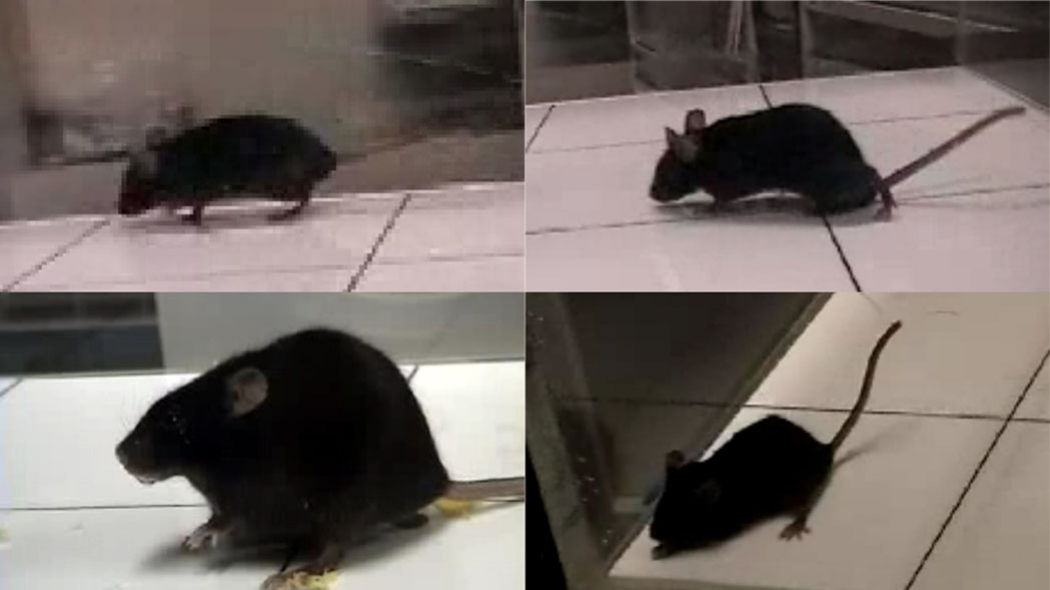
**Visible seizures observed in the M100200 mutant.** Upper left, normal gait; upper right, ataxic gait; lower left, convulsive seizure; lower right, lowered posture.

**Describe what you think is the most significant challenge impacting your research at this time and how will this be addressed over the next 10 years?**

TF: Virtual reality (VR) technology has been implemented in mouse behavioral phenotype analysis. VR technology not only enables various expansive experiments in a limited area but also enables brain-functional imaging or optogenetic experiments simultaneously with behavioral analyses. In addition, it may contribute to the studies which investigate the effects of VR experiences on human brain function, experiments which are difficult to conduct in humans.

“I think that one important thing is to develop and collaborate with different biological fields.” – *Hiroshi Mizuma*

HM: I think that one important thing is to develop and collaborate with different biological fields. For instance, it is becoming necessary that, to further advancement of bioimaging scanners, a deep understanding of brain function using small mammalian animals is needed. To overcome this, I am very interested in cutting-edge technologies to detect radiation in the universe, a field of astrophysics. By using these technologies, it will be possible to have high spatial resolution and to measure multiple nuclides simultaneously, giving insight into the complexity and interaction of brain functions.

**What changes do you think could improve the professional lives of early-career scientists?**

TF: Experienced scientists have knowledge on how to survive as scientists. I recommend early-career scientists collaborate with scientists who have long, successful careers.

“Experienced scientists have knowledge on how to survive as scientists.” – *Tamio Furuse*

HM: It is important to take on research which concerns not only your field but also other scientific fields. You never know how your skills are useful in other fields. I think interacting with as many researchers as possible is one way to expand your own potential.

**What's next for you?**

TF: To develop a novel methodology to integrate multiple methodologies which can monitor mouse brain function.

HM: I would like to challenge the innovative technologies involved in *in vivo* brain imaging based on a new interdisciplinary concept.
